# How Eudaimonia Impacts Global and Differential Life Satisfaction Independent of the General Mental Health Status

**DOI:** 10.3390/ijerph23030333

**Published:** 2026-03-06

**Authors:** Christopher Arnold, Beate Muschalla

**Affiliations:** Department of Psychotherapy and Diagnostics, Technische Universität Braunschweig, Humboldtstraße 33, 38106 Braunschweig, Germany; b.muschalla@tu-braunschweig.de

**Keywords:** eudaimonia, life satisfaction, well-being, mental health, resources, resilience

## Abstract

**Highlights:**

**Public health relevance—How does this work relate to a public health issue?**

- Global and differential life satisfaction are an important health indicator.
- Eudaimonic attitudes may contribute to life satisfaction and coping with mental health problems.

**Public health significance—Why is this work of significance to public health?**

- Overall, 30% of the general population suffers from mental health problems.
- Coping with mental health problems may benefit from high eudaimonia.

**Public health implications—What are the key implications or messages for practitioners, policy makers and/or researchers in public health?**

- Training eudaimonic attitudes may be useful in public health interventions.
- Eudaimonia could be considered as an outcome in intervention studies.

**Abstract:**

Growing evidence highlights a rather long-term perspective on well-being. Eudaimonia—living meaningfully, acting in accordance with one’s values, and accepting hardship in pursuit of worthwhile goals—is associated with better mental and physical health, resilience, and higher global life satisfaction. However, there is a lack of evidence investigating eudaimonia and its connection to satisfaction with specific life domains. This study explores how eudaimonia relates to global and domain-specific life satisfaction. A convenience general population sample (*N* = 394) was investigated by online questionnaire, assessing sociodemographic data, eudaimonia, health impairments, well-being, and satisfaction across 17 life domains with the Differential Life Burden Scale. High eudaimonia was associated with higher psychological well-being and work participation compared to individuals reporting low eudaimonia. Thereby people with high eudaimonia despite mental health problems reported higher satisfaction than those with mental health problems and low eudaimonia. People with high eudaimonia despite mental problems were similarly satisfied like people without mental problems but lower eudaimonia. For both global and domain-specific life satisfaction, individuals with and without mental health problems benefit from higher eudaimonia. Eudaimonia can be a valuable resource for mental health, overall life satisfaction, and satisfaction across various life domains.

## 1. Introduction

### 1.1. Hedonia and Eudaimonia

Recent decades of research on life satisfaction have ultimately largely focused on well-being and happiness, on the reduction in suffering, and the maximization of positive affects [1,2]. This concept is defined under the term “hedonia” [3]. Hedonia largely aims at immediate gratification instead of long-term benefit [4]. According to psychological hedonism, the goal or desire is to maximize pleasure and minimize pain. Good is what feels good. Actions are aimed at achieving and maintaining pleasure. Human behavior seeks to maintain the maximum positive ratio of pleasure and pain. It is argued that psychological hedonism shapes human behavior. Research on life satisfaction is often argued to be in connection with hedonia [4,5,6].

A largely contrasting approach to well-being and way of living is “eudaimonia”, which has been discussed since ancient times. For philosophers such as Socrates, Plato, and Xenophon, eudaimonia was already an important topic of discussion [7]. Eudaimonia has also become increasingly relevant in the research of recent decades. In contrast to hedonia, eudaimonia is not aimed at maximizing pleasure and minimizing discomfort, but rather understands well-being as a successful way of living or “mastery of life” [3,8]. This includes striving for good deeds, not least in the sense of being “good” for society. Among other things, conscious action that is in harmony with one’s own values and the pursuit of meaningfulness are important. In contrast to hedonia, eudaimonia adopts a more long-term or enduring perspective on well-being. It is not only the achievement of goals that contributes to fulfillment, but in particular the efforts to reach a defined goal. Ultimately, this also means enduring or even seeking out negativity or pain in order to pursue goals that are meaningful, good, and fulfilling [9]. According to the eudaimonic principle, a good life is an authentic and meaningful life, even if it involves suffering [8,10]. A mother who is woken up by her child crying loudly at 2 a.m. in the night will not experience any short-term hedonistic pleasure. However, the same situation can lead to high eudaimonic satisfaction because, despite the pain and suffering, it is seen as meaningful, as something that is right, and serves a higher purpose in accordance with eudaimonia. As this example shows, the level of hedonic and eudaimonic well-being can vary greatly in the same situation.

### 1.2. Benefit of Eudaimonia

Primarily in the last three decades, research on life satisfaction has increasingly broadened the focus to involve the influence of eudaimonia on life satisfaction and general well-being [3,10]. Empirical research suggests a positive effect of eudaimonia on general well-being and health [11,12,13]. This effect has been observed for both mental and physical health [14,15,16,17,18,19]. Eudaimonia is a facet of wisdom and is also observed in connection to resilience [20,21,22].

Eudaimonia is said to benefit general life satisfaction, and recent empirical studies have also shown the positive correlation of eudaimonia and life satisfaction in different populations [6,23,24,25,26].

The pursuit or experience of virtue, personal growth, and meaning, i.e., eudaimonia, and life satisfaction correlate positively. As discussed and demonstrated above, eudaimonia is often investigated in connection to global life satisfaction, i.e., a general evaluation of subjective satisfaction with one’s own life.

However, while eudaimonia may be present in one area of life, other domains of life may lack a sense of meaning, self-realization, or virtuousness, and therefore lack eudaimonia. Recent literature has shown that, in addition to general life satisfaction, satisfaction with important domains of life (e.g., interpersonal relationships, work, finances) can also correlate with eudaimonia [1,27,28,29,30].

### 1.3. Differential Life Satisfaction

The most widespread approach to life satisfaction is a global definition and measurement. Examples of the measurement of global life satisfaction include arguably the most widely used “Satisfaction with Life Scale” (SWLS) [31,32]. While such a global measurement can be used to comprehensively record subjective overall satisfaction, this does not provide any information about the extent to which a person is satisfied in specific life domains (which can be different). For this reason, life satisfaction should also be understood and measured differentially. Explicitly exploring domain-specific life satisfaction allows us to understand individual domains of life. In accordance with appraisal and subjective well-being [33,34], global life satisfaction can also prevail even though some domains of life are rated negatively. While global and differential life satisfaction may correlate, they are two different measurements with different purposes. Instruments for measuring differential life satisfaction, such as the Differential Life Burden Scale (DLB scale) [35], can be used for this intention.

### 1.4. Eudaimonic Behavior Contributes to Life Satisfaction

The literature on eudaimonia in relation to global life satisfaction is extensive and suggests a positive correlation [6,23,24,25,26]. Eudaimonic ideas and values can also be an important coping strategy in the treatment of mental illness [17,18,19,20,21], in the sense of accepting feeling unwell in the short-term in order to reach longer-lasting health-related or socially valuable goals. In this sense, eudaimonic behavior may account for life satisfaction despite episodes of feeling unwell. These connections between eudaimonia, life satisfaction, and mental illness are often discussed in association with resilience. Regardless of related factors such as hedonic well-being, eudaimonic values and beliefs are thought to contribute to life satisfaction as a resilience factor [10,36]. In particular, resilience in the sense of coping with adverse life events should also be mentioned here, i.e., how satisfaction is maintained or achieved despite negative circumstances (in relation to critical life events, mental illness), in the sense of post-traumatic growth [37]. For example, people with high eudaimonia frame adverse events to be transformative [38], meaning how they suffer but gain positive developments in the long run: for example, new insights about themselves, intrinsic growth, and relationships. Thus, people with a high eudaimonic orientation also experience critical life events, but often evaluate them differently, which in turn can be classified as a helpful coping strategy. In terms of dealing with adverse events, eudaimonia thus manifests itself both as a coping strategy and as a resilience factor.

The vast majority of the literature focuses on eudaimonia in relation to overall life satisfaction. Eudaimonia, or a eudaimonic way of life, can also be dependent on specific domains of life and can have varying degrees of impact on different domains of life. As shown in previous research, there is evidence that eudaimonia is also associated with domain-specific satisfaction, e.g., interpersonal or work-related domains [1,27,28,29,39].

To date, there is a lack of comprehensive and primarily empirical data examining the relationship between eudaimonia and differential, i.e., domain-specific, satisfaction. To our knowledge, there is currently no empirical data that examines the relationship between eudaimonia and several important domains of life and allows for comparisons between these domains. To fill this research gap, the objective of this study is to examine the extent to which eudaimonia, besides the mental health status, is related to global and differential life satisfaction.

The research questions are the following:

1. What characterizes people with high/low eudaimonia and with/without mental health problems in terms of socio-demographics, general wellbeing and functional health?
2. How satisfied with life (in terms of global and differential life satisfaction) are people with high/low eudaimonia and with/without mental health problems?

## 2. Methods

### 2.1. Study Design and Participants

This cross-sectional study was conducted using an online questionnaire between June 2024 and January 2025. An invitation for participation in the study was distributed to the general population in various ways in several German cities or via the internet (flyers, email distribution lists, social media, etc.). The sample consists of 394 people from the general public of Germany. Every person over the age of 18 was eligible to participate. The aim was to reach a heterogenous, population-near sample (all sexes, age groups, different professional and social backgrounds).

### 2.2. Data Collection Procedure

After an initial declaration of consent for participation in the study, the actual data collection followed. The data collection included a scale for eudaimonia (QEWB), the assessment of health impairments (IMET), general well-being (WHO-5), and differential life satisfaction (DLB scale). The data collection also included socio-demographic data (date of birth, sex, marital status, school and vocational qualifications, inability to work, unemployment, income, and mental health status. For categories, see Table 1).

### 2.3. Mental Health Problems

We asked participants, “How many weeks have you been unable to work (“on sick leave”) in the last 12 months?” Afterwards, data on psychological distress were collected. We asked participants, “Mental illnesses are widespread diseases. A quarter of the general population suffers from some kind of mental illness. Do you suffer or have you suffered from health problems lasting several weeks that are not of a purely physical nature?”, “Have you received any treatment for the above-mentioned or psychological complaints?”, and “Do the above-mentioned or psychological complaints regularly impair your way of living?” Participants could answer these questions with a “yes” or “no”. This question broadly explores whether any kind of clinically relevant mental health problem is present. This short exploration has been validated and used in earlier studies [40].

If participants indicated to suffer from mental health problems and that their life was impaired by these complaints, they were classified as a participant “with mental health problems (MentProb)” in the analysis.

### 2.4. Well-Being WHO-5

The WHO-Five Well-Being Index (WHO-5) [41,42] is a short questionnaire for the assessment of general well-being and psychological well-being. The WHO-5 starts with “Please indicate for each of the five statements which is closest to how you have been feeling over the last two weeks.” and continues with five statements such as “I have felt cheerful and in good spirits”, which are self-assessed on a six-point scale ranging from “all of the time” to “at no time”. Ratings can be given on a six-point Likert scale from “1 = all the time” to “6 = never”. The internal consistency of the WHO-5 in the current study is good, with a Cronbach’s alpha of = 0.84.

### 2.5. Eudaimonic Well-Being QEWB

The Questionnaire of Eudaimonic Wellbeing [22,43] is a self-rating scale for the assessment of well-being in accordance with the conceptualization in eudaimonic philosophy. According to the authors, the QEWB assesses eudaimonic aspects such as self-discovery, perceived development of one’s best potentials, a sense of purpose and meaning in life, intense involvement in activities, investment of significant effort, and enjoyment of activities as personally expressive. The QEWB starts with the statement, “This questionnaire contains a series of statements that refer to how you may feel things have been going in your life. Read each statement and decide the extent to which you agree or disagree with it. Try to respond to each statement according to your own feelings about how things are actually going, rather than how you might wish them to be.” The scale includes 21 items, which are rated on a five-point scale ranging from “1 = strongly disagree” to “5 = strongly agree”. The authors suggest a normal distribution of sum scores in the original samples. Sex and age are not evenly distributed in terms of the eudaimonia score [43]. To our knowledge, there is no established cut-off value for subdividing different levels of eudaimonia. Therefore, the sample-specific cut-off value used in this present study is the mean of the overall average. The mean is used to subdivide the sample into the two groups: people with “below-average” and “above-average” levels of eudaimonia.

For reliability analysis, Cronbach’s alpha was calculated to assess the internal consistency of the scale. In this study, the internal consistency of the questionnaire is satisfying, with a Cronbach’s alpha of = 0.88.

### 2.6. Participation Impairment IMET

The Index for the Assessment of Health Impairments (IMET) [44] is a self-rating for the assessment of participation and involvement according to the International Classification of Functioning, Disability and Health (ICF) [45]. The questionnaire is based on a holistic thinking model, as it is the basis for the ICF. In total, nine domains of life are self-assessed. Each item is further explained by a short annotation. The nine domains of life are: “usual activities of daily living”, “family and domestic obligations”, “errands outside the home”, “daily tasks and obligations”, “relaxation and leisure”, “social activities”, “close personal relationships”, “sex life”, and “stress and extraordinary burdens”. Answers for the first eight items are given on an eleven-point scale ranging from “0” (“no impairment”) to “10” (“no more activity possible”). The last area of life, “stress and extraordinary burdens”, is rated on an eleven-point scale ranging from “0” (“can withstand stress”) to “10” (“can no longer tolerate stress”). The IMET had a high Cronbach’s alpha of 0.90–0.91 in earlier studies [44], indicating that people perceive illness-related impairments across life domains. The internal consistency of the IMET in the present study is again excellent at a Cronbach’s alpha of = 0.93.

### 2.7. Differential Life Burden or Satisfaction DLB Scale

The Differential Life Burden Scale (DLB Scale) [35] is a self-assessment instrument for the rating of negative and positive emotions towards specific domains of life. The questionnaire consists of 17 items and starts with the statement: “Below you will find a list of important domains of life. Please indicate with a cross which of the following answers most closely match your feelings when you think of the corresponding area of life. Please do not leave out a single line!”. Afterwards, the following domains of life a rated: “partnership/marriage”, “sexuality”, “children”, “parents”, “friends”, “neighbors/acquaintances”, “colleagues”, “work”, “leisure”, “health”, “finances”, “residence”, “environment”, “homeland”, “politics”, “future”, and “life balance”. Responses are given on a six-point scale ranging from “1 = very negative”, “2 = negative”, “3 = slightly negative”, “4 = slightly positive”, “5 = positive”, to “6 = very positive”. The positive pole indicates satisfaction in the respective life domain. The completion time for the DLB self-rating is approximately three minutes. There were low to medium correlations between the items, and the scale showed moderate internal consistency in the original publication study (Cronbach’s alpha = 0.74–0.77) [35]. The moderate but not very high correlations indicate that the items measure something common (i.e., satisfaction) but also address different aspects (i.e., different life domains). The internal consistency of the questionnaire in the current study is Cronbach’s alpha = 0.86.

### 2.8. Statistics

Statistical analyses were conducted using IBM SPSS Statistics© (version 30.0.0.0; 64-bit). For group comparisons, chi-squared tests, one-way ANOVAs on ranks, or ANOVAs were performed, depending on the scale of measurement of the data. An alpha level of 0.05 was used for all statistical tests. Bonferroni correction was used for all post hoc analyses.

## 3. Results

### 3.1. Groups with High and Low Eudaimonia and With or Without Mental Health Problems

A total of *N* = 394 participants took part in the online survey.

The total mean score of the eudaimonic well-being according to the QEWB was *M* = 3.71 (*SD* = 0.5). The total mean score was used to divide the sample participants into “people with low eudaimonia (M < 3.71)” and “people with high eudaimonia (M ≥ 3.71)”.

These dichotomous classifications (low–high eudaimonia; with mental health problems—without mental health problems) were used to categorize the participants into four groups:

- Low eudaimonia + without mental health problem (*N* = 81) (LowEudai-NoMentProb).
- High eudaimonia + without mental health problem (*N* = 185) (HighEudai- NoMentProb).
- Low eudaimonia + mental health problem (*N* = 73) (LowEudai-MentProb).
- High eudaimonia + mental health problem (*N* = 55) (HighEudai-MentProb).

### 3.2. Sociodemographic Characteristics

The average age of all participants was 35.45 years (range 18–84 years) (*SD* = 17.04). Most participants indicated female sex (72.6%), followed by male (26.4%), and only 1% diverse. Sex distribution (only “female”, “male”) and school-leaving degree (>80% A-Levels “Abitur”) were not significantly different between the groups.

Other sociodemographic data, meaning marital status, partnership status, number of children, work time, and own and household income, showed some different distributions. The HighEudai-NoMentProb group was more often in a current relationship and had the highest average own and household income. Further detailed demographics can be found in Table 1.

### 3.3. Mental Health Problems and Eudaimonia

When comparing the four groups’ characteristics, consistent patterns emerge across all health-related and life satisfaction domains (Table 2 and Table 3):

Firstly, respondents without mental health problems and with high levels of eudaimonia gave the most positive ratings in all domains: high well-being, low impairment (Table 2), and high life satisfaction (Table 3).

**Table 1:** Sociodemographic data were divided into the eudaimonia and psychological distress groups (*N* = 394).

Variable		LowEudai-NoMentProb (LN) *N* = 81	HighEudai-NoMentProb (HN) *N* = 185	LowEudai-MentProb (LP) *N* = 73	HighEudai-MentProb (HP) *N* = 55	All (*N* = 394)		Post Hoc
		M (*SD*)	M (*SD*)	M (*SD*)	M (*SD*)	M (*SD*)		
Age		28.53 (14.26)	39.44 (18.14)	32 (14.14)	36.76 (16.72)	35.45 (17.04)	*F* (3, 390) = 9.527, *p* ≤ 0.001	LN vs. HN (*p* < 0.001, *M*_Diff_ = −10.9, 95%-CI [−16.75, −5.08]) LN vs. HP (*p* = 0.027, *M*_Diff_ = −8.23, 95%-CI [−15.88, −0.58]) HN vs. LP (*p* = 0.007, *M*_Diff_ = 7.44, 95%-CI [1.39, 13.5])
Female (“Sex” only female, male)		77.8%	68.6%	77.5%	77.4%	73.3%	*χ*^2^ (3) = 3.95, *p* = n.s.	
Marital status							*χ*^2^ (12) = 46.59, *p* < 0.001	
	Married + living together	13.6%	37.8%	12.7%	20.8%	25.9%		
	Married + living separately	0%	1.6%	1.4%	5.7%	1.8%		
	Unmarried	84%	54.1%	80.3%	58.5%	65.6%		
	Divorced	1.2%	5.9%	4.2%	11.3%	5.4%		
	Widowed	1.2%	0.5%	1.4%	3.8%	1.3%		
Partnership		38.3%	53.5%	33.8%	37.7%	44.6%	*χ*^2^ (3) = 11.62, *p* = 0.009	
No children		96.3%	75.7%	87.7%	80%	82.7%	*F* (3, 386) = 5.4, *p* < 0.001	
School-leaving qualification							*χ*^2^ (18) = 23.55, *p* = n.s.	
	Pupil at a general school	0%	0.5%	0%	0%	0.3%		
	Left school without a lower secondary school leaving certificate	0%	0%	1.4%	0%	0.3%		
	Lower secondary school leaving certificate (or former 8th grade school)	1.2%	0.5%	4.1%	0%	1.3%		
	Intermediate school leaving certificate/secondary school leaving certificate	3.7%	6.5%	11%	12.7%	7.6%		
	10th-grade polytechnic secondary school leaving certificate	1.2%	0.5%	1.4%	0%	0.8%		
	Vocational school certificate (without recognition as a university of applied sciences degree)	0%	3.2%	0%	3.6%	2%		
	General or subject-specific higher education entrance qualification or “Abitur”	93.8%	88.7%	82.1%	83.7%	87.2%		
Work time							*χ*^2^ (36) = 54.69, *p* = 0.024	
	Full-time employed with a weekly working time of 35 h or more	14.8%	33.5%	15.1%	25.5%	25.1%		
	Part-time employed with a weekly working time of 15 to 34 h	16%	11.9%	9.6%	14.5%	12.7%		
	Part-time or hourly employed with a weekly working time of less than 15 h	9.9%	6.5%	8.2%	3.6%	7.1%		
	Federal voluntary service, social or ecological year, or military service	0%	0%	1.4%	0%	0.3%		
	On maternity/parental leave	0%	1.1%	0%	1.8%	0.8%		
	On the other leave of absence	0%	0.5%	0%	1.8%	0.5%		
	Currently unemployed/in 0 short-term work	1.2%	0%	0%	3.6%	1.3%		
	Disability pension	1.2%	2.2%	8.2%	3.6%	3.3%		
	Retirement pension	3.7%	8.1%	4.1%	3.6%	5.8%		
	Not working	3.7%	0.5%	4.1%	0%	1.8%		
	Housewife/-husband	0%	0%	1.4%	0%	0.3%		
	In vocational training (including technical schools for industrial professions)	0%	1.1%	1.4%	1.8%	1%		
	In school education (including university and college)	49.4%	33.5%	46.6%	40%	40.1%		
Own income (categories)							*H* (3) = 25.73, *p* < 0.001	LN vs. HN (*Rank_Diff_* = −65.36, *p* < 0.001) LP vs. HN (*Rank_Diff_* = 59.19, *p* < 0.001)
	Up to under €500	27.2%	16.8%	9.6%	14.5%	19.3%		
	€500–under €650	14.8%	8.1%	5.5%	5.5%	8.9%		
	€650–under €750	3.7%	1.1%	0%	1.8%	1.5%		
	€750–under €900	7.4%	2.7%	11%	3.6%	5.6%		
	€900–under €1000	4.9%	4.9%	2.7%	5.5%	5.6%		
	€1000–under €1150	3.7%	3.8%	13.7%	14.5%	6.6%		
	€1150–under €1250	3.7%	2.2%	1.4%	7.3%	4.6%		
	€1250–under €1500	4.9%	8.1%	2.7%	7.3%	7.1%		
	€1500–under €2000	8.6%	9.2%	13.7%	12.7%	10.2%		
	€2000–under €2500	7.4%	8.6%	15.1%	16.4%	8.9%		
	€2500–under €3500	9.9%	18.4%	11%	5.5%	12.4%		
	€3500–under €5000	3.7%	8.6%	6.8%	3.6%	5.3%		
	€5000 and more	0%	7.6%	6.8%	1.8%	4.1%		
Household income (categories)							*H* (3) = 26.44, *p* < 0.001	LN vs. HN (*Rank_Diff_* = −51.61, *p* = 0.004) LP vs. HN (*Rank_Diff_* = 71.9, *p* < 0.001)
	Up to under €500	13.6%	8.1%	9.6%	5.5%	9.1%		
	€500–under €650	3.7%	1.6%	5.5%	3.6%	3%		
	€650–under €750	3.7%	1.6%	0%	0%	1.5%		
	€750–under €900	4.9%	3.2%	11%	5.5%	5.3%		
	€900–under €1000	4.9%	3.2%	2.7%	1.8%	3.3%		
	€1000–under €1150	4.9%	5.9%	13.7%	12.7%	8.1%		
	€1150–under €1250	4.9%	2.2%	1.4%	7.3%	3.3%		
	€1250–under €1500	6.2%	2.7%	2.7%	3.6%	3.6%		
	€1500–under €2000	9.9%	4.3%	13.7%	7.3%	7.6%		
	€2000–under €2500	2.5%	6.5%	15.1%	20%	9.1%		
	€2500–under €3500	12.3%	13%	11%	1.8%	10.9%		
	€3500–under €5000	13.6%	20%	6.8%	20%	16.2%		
	€5000 and more	14.8%	27.6%	6.8%	10.9%	18.8%		

Note. M: mean value; SD: standard deviation; n.s. = not significant.

**Table 2:** Health data were divided into the eudaimonia and psychological distress groups (*N* = 394).

Variable	LowEudai-NoMentProb (LN) *N* = 81	HighEudai-NoMentProb (HN) *N* = 185	LowEudai-MentProb (LP) *N* = 73	HighEudai-MentProb (HP) *N* = 55	All (*N* = 394)		Post Hoc
	M (*SD*)	M (*SD*)	M (*SD*)	M (*SD*)	M (*SD*)		
In treatment for mental health problems	11.1%	15.1%	84.9%	85.5%	37.1%	*χ*^2^ (3) = 18.91, *p* < 0.001	LN < LP LN < HP HN < LP HN < HP
Well-being WHO-5 Scale from 1 = all the time to 6 = never	3.49 (0.85)	2.96 (0.84)	4.35 (0.84)	3.88 (0.85)	3.45 (1)	*F* (3, 390) = 53.69, *p* < 0.001	LN vs. HN (*p* < 0.001, *M*_Diff_ = 0.53, 95%-CI [0.23, 0.83]) LN vs. LP (*p* < 0.001, *M*_Diff_ = −0.88, 95%-CI [−1.24, −0.52]) HN vs. LP (*p* < 0.001, *M*_Diff_ = −1.41, 95%-CI [−1.72, −1.1]) HN vs. HP (*p* < 0.001, *M*_Diff_ = −0.88, 95%-CI [−1.22, −0.53]) LP vs. HP (*p* = 0.003, *M*_Diff_ = 0.53, 95%-CI [0.14, 0.93])
Inability to work (weeks in the last 12 months)	1.51 (5.91)	1.27 (2.33)	3.11 (8.79)	3.17 (7.13)	1.92 (5.61)	*F* (3, 388) = 2.99, *p* = 0.031	
Unemployment (amount over lifetime)	0.35 (0.76)	0.44 (0.71)	1.62 (5.36)	0.91 (1.57)	0.7 (2.48)	*F* (3, 390) = 4.82, *p* = 0.003	LN vs. LP (*p* = 0.008, *M*_Diff_ = −1.27, 95%-CI [−2.32, −0.22]) HN vs. LP (*p* = 0.003, *M*_Diff_ = −1.18, 95%-CI [−2.08, −0.28])
Eudaimonia scale Scale from 1 = fully disagree to 5 = fully agree	3.33 (0.29)	4.04 (0.24)	3.08 (0.44)	4.01 (0.21)	3.71 (0.5)	*F* (3, 390) = 257.8, *p* < 0.001	LN vs. HN (*p* < 0.001, *M*_Diff_ = −0.72, 95%-CI [−0.82, −0.61]) LN vs. LP (*p* < 0.001, *M*_Diff_ = 0.24, 95%-CI [0.12, 0.37]) LN vs. HP (*p* < 0.001, *M*_Diff_ = −0.68, 95%-CI [−0.82, −0.55]) HN vs. LP (*p* < 0.001, *M*_Diff_ = 0.96, 95%-CI [0.85, 1.07]) LP vs. HP (*p* < 0.001, *M*_Diff_ = −0.92, 95%-CI [−1.06, −0.79])
Impairments IMET Scale from 0 = no impairment to 10 = no more activity possible	1.92 (2.02)	1.31 (1.65)	4.24 (1.83)	3.26 (2.03)	2.25 (2.14)	*F* (3, 390) = 52.37, *p* < 0.001	LN vs. LP (*p* < 0.001, *M*_Diff_ = −2.32, 95%-CI [−3.1, −1.54]) LN vs. HP (*p* < 0.001, *M*_Diff_ = −1.33, 95%-CI [−2.18, −0.49]) HN vs. LP (*p* < 0.001, *M*_Diff_ = −2.94, 95%-CI [−3.6, −2.27]) HN vs. HP (*p* < 0.001, *M*_Diff_ = −1.95, 95%-CI [−2.69, −1.21]) LP vs. HP (*p* = 0.015, *M*_Diff_ = 0.98, 95%-CI [0.12, 1.85])

Note. M: mean value; SD: standard deviation.

**Table 3:** Global and differential life satisfaction (DLB scale) was divided into the eudaimonia and psychological distress groups (*N* = 394).

Variable	LowEudai-NoMentProb (LN) *N* = 81	HighEudai-NoMentProb (HN) *N* = 185	LowEudai-MentProb (LP) *N* = 73	HighEudai-MentProb (HP) *N* = 55	All (*N* = 394)		Post Hoc
	M (*SD*)	M (*SD*)	M (*SD*)	M (*SD*)	M (*SD*)		
Global life satisfaction (DLB scale mean) Scale from 1 very negative to 6 = very positive	4.05 (0.53)	4.39 (0.48)	3.34 (0.61)	3.89 (0.58)	4.06 (0.66)	*F* (3, 390) = 71.58, *p* < 0.001	LN vs. HN (*p* < 0.001, *M*_Diff_ = −0.35, 95%-CI [−0.53, −0.16]) LN vs. LP (*p* < 0.001, *M*_Diff_ = 0.71, 95%-CI [0.48, 0.94]) HN vs. LP (*p* < 0.001, *M*_Diff_ = 1.06, 95%-CI [0.86, 1.25]) HN vs. HP (*p* < 0.001, *M*_Diff_ = 0.5, 95%-CI [0.28, 0.71]) LP vs. HP (*p* < 0.001, *M*_Diff_ = −0.56, 95%-CI [−0.81, −0.31])
Partnership/marriage	4.28 (1.39)	4.91 (1.12)	3.37 (1.47)	4.4 (1.41)	4.43 (1.41)	*F* (3, 390) = 25.44, *p* < 0.001	LN vs. HN (*p* = 0.002, *M*_Diff_ = −0.63, 95%-CI [−1.09, −0.17]) LN vs. LP (*p* < 0.001, *M*_Diff_ = 0.91, 95%-CI [0.36, 1.47]) HN vs. LP (*p* < 0.001, *M*_Diff_ = 1.54, 95%-CI [1.07, 2.02]) LP vs. HP (*p* < 0.001, *M*_Diff_ = −1.03, 95%-CI [−1.64, −0.42])
Sexuality	4.28 (1.24)	4.50 (1.1)	3.14 (1.39)	3.71 (1.42)	4.09 (1.34)	*F* (3, 390) = 23.79, *p* < 0.001	LN vs. LP (*p* < 0.001, *M*_Diff_ = 1.15, 95%-CI [0.62, 1.68]) LN vs. HP (*p* = 0.048, *M*_Diff_ = 0.58, 95%-CI [0.00, 1.15]) HN vs. LP (*p* < 0.001, *M*_Diff_ = 1.37, 95%-CI [0.91, 1.82]) HN vs. HP (*p* < 0.001, *M*_Diff_ = 0.79, 95%-CI [0.29, 1.3])
Children	4.25 (1.26)	4.76 (1.22)	3.58 (1.55)	4.25 (1.38)	4.36 (1.38)	*F* (3, 390) = 14.57, *p* < 0.001	LN vs. HN (*p* = 0.023, *M*_Diff_ = −0.51, 95%-CI [−0.97, −0.04]) LN vs. LP (*p* = 0.01, *M*_Diff_ = 0.67, 95%-CI [0.11, 1.23]) HN vs. LP (*p* < 0.001, *M*_Diff_ = 1.18, 95%-CI [0.7, 1.66]) LP vs. HP (*p* = 0.024, *M*_Diff_ = −0.68, 95%-CI [−1.3, −0.06])
Parents	4.59 (1.21)	4.57 (1.28)	3.52 (1.42)	3.49 (1.56)	4.23 (1.43)	*F* (3, 390) = 18.27, *p* < 0.001	LN vs. LP (*p* < 0.001, *M*_Diff_ = 1.07, 95%-CI [0.5, 1.65]) LN vs. HP (*p* < 0.001, *M*_Diff_ = 1.1, 95%-CI [0.48, 1.72]) HN vs. LP (*p* < 0.001, *M*_Diff_ = 1.05, 95%-CI [0.56, 1.54]) HN vs. HP (*p* < 0.001, *M*_Diff_ = 1.08, 95%-CI [0.53, 1.62])
Friends	5.06 (0.97)	5.1 (0.82)	4.38 (1.1)	4.96 (0.98)	4.94 (0.96)	*F* (3, 390) = 10.99, *p* < 0.001	LN vs. LP (*p* < 0.001, *M*_Diff_ = 0.68, 95%-CI [0.28, 1.08]) HN vs. LP (*p* < 0.001, *M*_Diff_ = 0.71, 95%-CI [0.37, 1.05]) LP vs. HP (*p* = 0.003, *M*_Diff_ = −0.58, 95%-CI [−1.02, −0.14])
Neighbors/acquaintances	4.07 (0.86)	4.35 (0.88)	3.66 (0.99)	4.38 (1.01)	4.17 (0.95)	*F* (3, 390) = 11.11, *p* = 0.004	LN vs. LP (*p* = 0.031, *M*_Diff_ = 0.42, 95%-CI [0.02, 0.81]) HN vs. LP (*p* < 0.001, *M*_Diff_ = 0.69, 95%-CI [0.35, 1.02]) LP vs. HP (*p* < 0.001, *M*_Diff_ = −0.72, 95%-CI [−1.16, −0.29])
Colleagues	4.2 (0.87)	4.48 (0.92)	3.71 (1.07)	4.35 (1.02)	4.26 (0.99)	*F* (3, 390) = 11.61, *p* < 0.001	LN vs. LP (*p* = 0.01, *M*_Diff_ = 0.49, 95%-CI [0.08, 0.89]) HN vs. LP (*p* < 0.001, *M*_Diff_ = 0.77, 95%-CI [0.42, 1.12]) LP vs. HP (*p* = 0.001, *M*_Diff_ = −0.63, 95%-CI [−1.08, −0.18])
Work	3.67 (1)	4.41 (1)	2.96 (1.25)	4 (1.26)	3.93 (1.21)	*F* (3, 390) = 32.83, *p* < 0.001	LN vs. HN (*p* < 0.001, *M*_Diff_ = −0.74, 95%-CI [−1.12, −0.35]) LN vs. LP (*p* < 0.001, *M*_Diff_ = 0.71, 95%-CI [0.24, 1.17]) HN vs. LP (*p* < 0.001, *M*_Diff_ = 1.45, 95%-CI [1.05, 1.85]) LP vs. HP (*p* < 0.001, *M*_Diff_ = −1.04, 95%-CI [−1.56, −0.53])
Leisure	4.98 (0.84)	5.23 (0.82)	4.36 (1.14)	4.75 (0.99)	4.95 (0.97)	*F* (3, 390) = 17.19, *p* < 0.001	LN vs. LP (*p* < 0.001, *M*_Diff_ = 0.62, 95%-CI [0.23, 1.01]) HN vs. LP (*p* < 0.001, *M*_Diff_ = 0.88, 95%-CI [0.54, 1.21]) HN vs. HP (*p* = 0.003, *M*_Diff_ = −0.49, 95%-CI [0.12, 0.86])
Health	4.20 (1.03)	4.39 (1.06)	3.16 (1.24)	3.6 (1.33)	4.01 (1.22)	*F* (3, 390) = 23.72, *p* < 0.001	LN vs. LP (*p* < 0.001, *M*_Diff_ = 1.03, 95%-CI [0.55, 1.52]) LN vs. HP (*p* = 0.016, *M*_Diff_ = 0.6, 95%-CI [0.07, 1.12]) HN vs. LP (*p* < 0.001, *M*_Diff_ = 1.23, 95%-CI [0.81, 1.64]) HN vs. HP (*p* < 0.001, *M*_Diff_ = 0.79, 95%-CI [0.33, 1.25])
Finances	3.69 (1.07)	4.3 (1.28)	3.1 (1.22)	3.38 (1.39)	3.82 (1.33)	*F* (3, 390) = 20.09, *p* < 0.001	LN vs. HN (*p* = 0.002, *M*_Diff_ = −0.61, 95%-CI [−1.05, −0.17]) LN vs. LP (*p* = 0.019, *M*_Diff_ = 0.6, 95%-CI [0.06, 1.13]) HN vs. LP (*p* < 0.001, *M*_Diff_ = 1.21, 95%-CI [0.75, 1.66]) HN vs. HP (*p* < 0.001, *M*_Diff_ = 0.92, 95%-CI [0.41, 1.43])
Residence	4.47 (1.14)	4.59 (1.24)	3.66 (1.34)	4.36 (1.18)	4.36 (1.27)	*F* (3, 390) = 10.43, *p* < 0.001	LN vs. LP (*p* < 0.001, *M*_Diff_ = 0.81, 95%-CI [0.29, 1.34]) HN vs. LP (*p* < 0.001, *M*_Diff_ = 0.94, 95%-CI [0.49, 1.39]) LP vs. HP (*p* = 0.008, *M*_Diff_ = −0.71, 95%-CI [−1.29, −0.12])
Environment	3.1 (1.02)	3.24 (1.11)	2.53 (1)	2.64 (1.13)	2.99 (1.11)	*F* (3, 390) = 9.93, *p* < 0.001	LN vs. LP (*p* = 0.007, *M*_Diff_ = 0.57, 95%-CI [0.11, 1.02]) HN vs. LP (*p* < 0.001, *M*_Diff_ = 0.7, 95%-CI [0.31, 1.10]) HN vs. HP (*p* = 0.002, *M*_Diff_ = 0.6, 95%-CI [0.16, 1.04])
Homeland	4.33 (1.15)	4.42 (1.21)	3.64 (1.28)	3.82 (1.35)	4.17 (1.27)	*F* (3, 390) = 8.9, *p* < 0.001	LN vs. LP (*p* = 0.003, *M*_Diff_ = 0.69, 95%-CI [0.16, 1.22]) HN vs. LP (*p* < 0.001, *M*_Diff_ = 0.77, 95%-CI [0.32, 1.22]) HN vs. HP (*p* = 0.01, *M*_Diff_ = 0.6, 95%-CI [0.1, 1.1])
Politics	2.23 (0.81)	2.52 (0.97)	2.08 (0.83)	2.27 (1.04)	2.35 (0.94)	*F* (3, 390) = 4.62, *p* = 0.003	HN vs. LP (*p* = 0.004, *M*_Diff_ = 0.44, 95%-CI [0.1, 0.78])
Future	3.44 (1.06)	4.18 (0.98)	2.73 (1.18)	3.64 (1.16)	3.68 (1.19)	*F* (3, 390) = 34.69, *p* < 0.001	LN vs. HN (*p* < 0.001, *M*_Diff_ = −0.73, 95%-CI [−1.11, −0.36]) LN vs. LP (*p* < 0.001, *M*_Diff_ = 0.72, 95%-CI [0.26, 1.17]) HN vs. LP (*p* < 0.001, *M*_Diff_ = 1.45, 95%-CI [1.06, 1.84]) HN vs. HP (*p* = 0.006, *M*_Diff_ = 0.54, 95%-CI [0.11, 0.97]) LP vs. HP (*p* < 0.001, *M*_Diff_ = −0.91, 95%-CI [−1.41, −0.41])
Life balance	3.93 (0.89)	4.72 (0.72)	3.12 (1.12)	4.2 (1.06)	4.19 (1.07)	*F* (3, 390) = 58.98, *p* < 0.001	LN vs. HN (*p* < 0.001, *M*_Diff_ = −0.79, 95%-CI [−1.11, −0.48]) LN vs. LP (*p* < 0.001, *M*_Diff_ = 0.8, 95%-CI [0.42, 1.18]) HN vs. LP (*p* < 0.001, *M*_Diff_ = 1.6, 95%-CI [1.27, 1.92]) HN vs. HP (*p* = 0.001, *M*_Diff_ = 0.52, 95%-CI [0.16, 0.88]) LP vs. HP (*p* < 0.001, *M*_Diff_ = −1.08, 95%-CI [−1.5, −0.65])

Note. M: mean value; SD: standard deviation.

Secondly, respondents with mental health problems and low levels of eudaimonia gave the worst ratings in all domains (Table 2 and Table 3).

Thirdly, the results of the group with mental health problems but high eudaimonia are of particular interest in comparison to individuals without mental health problems but low eudaimonia. Well-being, global, and differential life satisfaction were rated similarly by both groups (Table 2 and Table 3). Despite reporting mental health problems, individuals in the HighEudai-MentProb group showed overall and differential life satisfaction levels comparable to those of individuals without mental health problems but with low eudaimonia (LowEudai-NoMentProb). In 14 out of 17 life domains, there were no significant differences between the two groups in satisfaction. The same was true for overall life satisfaction (Table 3).

## 4. Discussion

To our knowledge, this is the first explorative study to examine the relationship between mental health, eudaimonia, and differential life satisfaction in detail. While previous studies have occasionally addressed the relationship between eudaimonia and different selected domains of life, the present data allow for a comparison of satisfaction levels across a wider range of life domains.

### 4.1. Mental Health, Well-Being, Satisfaction

Participants with mental health problems rate their psychological well-being (WHO-5) as the lowest and have more work-related problems than others. This confirms the widely researched correlation between psychological burden and low psychological well-being, as well as work-related participation problems [46,47,48].

However, findings show that, besides the mental health condition, additional aspects must be considered in order to understand why some individuals with mental health problems might feel better and less impaired: within those with mental health problems, individuals with high eudaimonia rated their psychological well-being higher than individuals with low eudaimonia. This suggests that eudaimonia may be both a protective factor in the event of mental health problems or an aspect of successful life coping in the sense of “mastery of life” [6,8,10,20,21,33,49]. It may be that those with higher eudaimonia (even if mentally ill) are better able to take the risks and hurdles of pursuing and fighting for positive goals. This may also apply to overcoming unemployment and searching for a new job.

A similar pattern is evident concerning differential life satisfaction. Mentally healthy participants reported the highest level of life satisfaction. This is consistent with other research findings [50,51,52]. Mental health problems are known to be associated with negatively experienced interpersonal relationships [53,54], and close partnerships [55,56]. How positively or negatively work is perceived can vary in the case of mental health problems, depending on whether the concrete work situation is a resource or a burden in the concrete case [57,58]. Mental health problems can also limit and alter the ability to reflect (“life balance”) and make future prospects (“future”), and thus impact the subjective evaluation of domains of life [33]. The generally lower life satisfaction in those with mental health problems is also reflected in specific domains of life.

### 4.2. Eudaimonia and Global Life Satisfaction

As expected from the known literature [50,51,52,59], higher global life satisfaction occurred in persons with higher eudaimonia and without mental health problems.

Similar to the findings on psychological well-being, eudaimonia was associated with even higher general life satisfaction, both in the presence and absence of mental health problems. The findings from the present study confirm previous literature regarding the association between eudaimonia and general life satisfaction. Besides the well-known impact of mental health problems, additional explanatory value of eudaimonia for global life satisfaction can be assumed.

### 4.3. Eudaimonia and Differential Life Satisfaction

In addition, the findings suggest eudaimonia is relevant for differential life satisfaction. In the life domains of way of living (leisure, future, life balance), living environment (environment, home), as well as sexuality, parents, health, and finance, it made no significant difference whether individuals had low or high eudaimonia. However, it was found that for most direct interpersonal relationships (partnership/marriage, children, friends, neighbors), the work environment (colleagues, work), and “residence”, high eudaimonia is associated with higher life satisfaction, especially in people with mental health problems. The differences in the group comparisons are between 0.5 and 1.5 scale points. For several domains of life (e.g., “life balance”), group differences are rather large. While in some life domains one group is rather satisfied, another group rates the same life domain more negatively, underlining the practical relevance of differentiated approaches to life satisfaction. For other life domains (e.g., “neighbors/acquaintances”), group differences are rather small, which also indicates that mental health and eudaimonia are less practically relevant for the degree of satisfaction.

While the findings suggest that eudaimonia is relevant for interpersonal and work-related domains, other more societal domains (“politics”, “environment”) seem less associated with eudaimonia. These domains, which are overall rated as comparatively dissatisfactory, also show low satisfaction in other studies [60]. Additional psychological aspects, such as “cognitively mediated suffering”, may contribute to negative attitudes towards these domains of life.

The findings lead to the hypothesis that eudaimonia may be a protective factor in a number of life domains and can come along with higher life satisfaction despite mental health problems. These results underline the importance of considering eudaimonia in diagnostics and interventions for people with mental health problems [8,61].

### 4.4. Limitations and Future Research

The cross-sectional design and the characteristics of the sample surveyed restrict the generalizability of the results. While the aim was to generate a sample that was as close as possible to the general population, the sample is over- and underrepresented in terms of several sample characteristics. This should be taken into account more strongly in future studies in order to ensure better generalizability. Due to the existing study characteristics, the generalizability of the current results is limited. However, since prototypical groups with defined characteristics (with/out mental health problems, high/low eudaimonia) were of interest for comparison here, the sample does not need to be fully population representative.

The dichotomized variables used for group definition limit the interpretability of the results and their generalizability. Future research should focus on continuous and stricter categorial approaches for both eudaimonia and mental health status (type of symptomatology, severity, impairments). Eudaimonia in particular should be understood as a continuous variable and therefore considered as such in future research.

The group-aggregated analysis cannot be automatically applied to single cases in clinical practice: Implications for practice in concrete cases require a more comprehensive assessment of mental health problems in type and illness-related impairments.

This present study provides initial quantitative evidence of the connection between eudaimonia and life satisfaction. Future studies should assess mental health status in a more differentiated way (e.g., differentiating mental health problems in affective, anxiety, or personality disorders). In treatment-prone groups, the usability of eudaimonia in skills training or psychotherapy should be tested.

## 5. Conclusions

While higher levels of eudaimonia are associated with higher domain-specific life satisfaction in nuclear family, work, and life perspective for people with and without mental health problems, people with mental health problems benefit most from eudaimonia. Despite mental health problems, a high level of eudaimonia achieves a similar level of satisfaction in most domains as compared to healthy people with low eudaimonia.

Eudaimonia can be a valuable resource for psychological health, overall life satisfaction, and satisfaction across various life domains in both individuals without, but mainly in people suffering from mental health problems.

## Data Availability

The data will be made available upon reasonable request by the corresponding author.
